# Probiotics, Prebiotics, and Synbiotics for the Prevention of Necrotizing Enterocolitis

**DOI:** 10.3389/fnut.2021.667188

**Published:** 2021-09-07

**Authors:** Kiera Murphy, R. Paul Ross, C. Anthony Ryan, Eugene M. Dempsey, Catherine Stanton

**Affiliations:** ^1^Food Biosciences Department, Teagasc Food Research Centre, Cork, Ireland; ^2^APC Microbiome Ireland, University College Cork, Cork, Ireland; ^3^Neonatal Intensive Care Unit, Department of Paediatrics and Child Health, University College Cork, Cork, Ireland

**Keywords:** microbiome, prebiotic, probiotic, synbiotic, necrotizing enterocolitis

## Abstract

Necrotizing enterocolitis (NEC) is a major cause of morbidity and mortality in preterm infants. The exact mechanism by which NEC develops is poorly understood however there is growing evidence to suggest that perturbations in the early-life gut microbiota composition increase the risk for NEC. Modulation of the gut microbiota with probiotics, prebiotics, or in combination (synbiotics) is an area which has attracted intense interest in recent years. In this narrative review, we present an overview of the role of the gut microbiota in the pathogenesis of NEC. We also examine the evidence currently available from randomized controlled trials, observational studies, systematic reviews, and meta-analysis examining the role of probiotics, prebiotics, and synbiotics in reducing the risk of or preventing NEC. Current clinical practice guidelines with recommendations on the routine administration of probiotics to preterm infants for NEC are also explored.

## Introduction

The early life gut microbiome is a dynamic community of microorganisms that play an important role in infant health. Factors influencing the development of the infant gut microbiota include mode of delivery (caesarean section vs. vaginal birth), gestational age (premature vs. full-term birth), antibiotic use, mode of feeding (formula vs. breastfeeding), and environmental factors (1, 2). *Bifidobacterium* typically dominate the microbiota in vaginally delivered, breastfed infants. Infants delivered by caesarean section are characterised by reduced *Bacteroides* and *Bifidobacterium* and increased colonization by opportunistic pathogens such as *Enterococcus, Enterobacter, Clostridium*, and *Klebsiella* species (1–5). Disrupted microbiota acquisition during this critical developmental window may have both short and long-term health implications. Imbalances in the composition of the gut microbiota have been associated with a wide range of diseases including allergic disorders, type 1 diabetes, inflammatory bowel disease, obesity, sepsis, and necrotizing enterocolitis (NEC) (6–10).

With our growing understanding of the role of the microbiome in health and disease, the use of probiotics to promote a healthy microbiome is an active area of research. Probiotics are defined by the FAO/WHO as “live microorganisms which when administered in adequate amounts confer a health benefit on the host” (11). Probiotics may positively contribute to host health by modulating immune responses such as inflammation, improving the function of the intestinal mucosal barrier, modulating the expression of host genes, and preventing colonization by pathogenic bacteria. One of the mechanisms through which probiotics influence a range of health parameters is through the production of bioactive compounds. Vitamins, antimicrobial peptides, conjugated linoleic acid (CLA), exopolysaccharides, gamma aminobutyric acid (GABA), and short-chain fatty acids (SCFAs) are all examples of microbially produced bioactive compounds (12). SCFAs including acetate, propionate, and butyrate are crucial for gut health and can modulate metabolic activity including colonocyte function, gut homeostasis, and the immune system (13). While CLA has immunomodulating properties, reducing the proinflammatory cytokines (14).

Prebiotics are defined as “a substrate that is selectively utilized by host microorganisms conferring a health benefit” (15). Prebiotics specifically stimulate the growth of beneficial microbes including bifidobacteria and lactobacilli. Prebiotics are naturally found in human milk (HM), which contains over 200 human milk oligosaccharides (HMOs) (16). HMOs can increase the proportion of HMO-consuming bifidobacteria and *Bacteroides* in breast-fed infants. Infant formula are now often supplemented with prebiotics and probiotics to mimic the functional effects of HMOs and HM bacteria (17). A synbiotic is defined as “a mixture comprising live microorganisms and substrate(s) selectively utilized by host microorganisms that confers a health benefit on the host” (18). A synbiotic may be classified as complementary or synergistic. In a complementary synbiotic the probiotic and prebiotic provide a health benefit together but are not co-dependent. In synergistic synbiotics, the prebiotic is chosen based on its ability to be selectively utilized by the probiotic.

One particular area that has produced positive results in probiotic intervention studies is the prevention of NEC in preterm infants (19). NEC is a serious acquired disease of the gastrointestinal tract, characterized by acute intestinal necrosis. The incidence rate of NEC is reported as approximately 5–10% of very preterm or very low birth weight (VLBW) infants (20, 21). A 2020 systematic review reported that seven out of 100 VLBW infants in the neonatal intensive care unit (NICU) are likely to develop NEC (22). The mortality rate is reported at 20–30%, and infants who survive NEC have a greater risk of neurodevelopmental delays (23). Preterm infants represent a particularly vulnerable group especially those weighing <1,500 g, VLBW infants, and <1,000 g, extremely low birth weight (ELBW).

## Infant Gut Microbiota and Necrotizing Enterocolitis

The pathogenesis of NEC is complex and the exact etiology remains unknown; however, immaturity of the intestinal barrier and immune system are thought to contribute (24). The intestinal microbiome is also believed to contribute to the pathogenesis of NEC. Experiments using animal models have shown that NEC does not occur in germ-free mice and toll-like receptor targeted knockout mice strongly suggesting that the gut microbiome is critical for NEC development (25–28). Studies using 16S rRNA gene sequencing have reported a reduction in microbial community diversity, decreases in Firmicutes, and an increase in Proteobacteria in the stool of NEC patients (29–31). Proteobacteria contain numerous gram-negative pathogens with high levels of lipopolysaccharide (LPS). TLR4 recognizes LPS and TLR4 activation leads to inhibition of mucosal repair. Breakdown of the gut barrier and translocation of pathogenic bacteria leads to an increased inflammatory response, resulting in NEC (32). Patients with NEC have been reported to have higher levels of LPS in their plasma (25). In addition, intraperitoneal injection of LPS to rats and mice has been demonstrated to induce intestinal injury and shock (33). Several studies have linked colonization by clostridia with NEC and pointed toward a potential deleterious role in the pathogenesis of NEC (34, 35). The exact mechanism is unclear but it is thought that lactose fermentation leading to an overproduction of butyric acid and the presence of toxin genes may play a role (36, 37).

Olm et al. performed metagenomic analysis of faecal samples from premature infants to identify microbial features predictive of NEC (38). Samples collected prior to NEC onset contained significantly higher *Klebsiella*, bacteria encoding fimbriae, and secondary metabolite gene clusters related to bacteriocin production and quorum sensing. Bacterial replication rates were measured from metagenomic data by determining the difference in DNA sequencing coverage between the origin and the terminus of replication. Replication rates, particularly that of Enterobacteriaceae, were significantly higher two days prior to NEC diagnosis. Microbiome analysis of faecal samples may not accurately represent the bacterial communities at the site of injury, the intestinal mucosa. A study by Romano-Keeler et al. examined the microbiome in both NEC tissue and faecal samples in surgical patients with and without NEC (39). The authors reported a tissue-specific overrepresentation of Firmicutes, specifically *Staphylococcus* and *Clostridium* and a lower abundance of *Actinomyces* and *Corynebacterium* in NEC.

EPIPAGE 2, a prospective cohort study in France, assessed nutritional strategies and the gut microbiota as risk factors for NEC (40). Slower rates of progression of enteral feeding and less favorable direct-breastfeeding policies were associated with a higher risk of NEC. An association between *Clostridium neonatale* and *Staphylococcus aureus* with NEC was also noted. Interestingly, no relation between antibiotic treatment and the onset of NEC was observed. This is in contrast to several studies which have reported that early antibiotic use in preterm infants increases the risk of NEC (41–43). Acid-suppressive medications such as histamine-2 receptor antagonists and proton pump inhibitors (PPIs) are routinely used for the treatment of upper gastrointestinal bleeding or gastroesophageal reflux in preterm infants. Exposure to these acid-suppressive medicines has been associated with an increased risk of NEC (44, 45). Changes in gut microbiota composition related to PPI therapy have been well-documented (46, 47). Feeding with HM provides beneficial bacteria and essential prebiotic substances including non-digestible HMOs, immunoprotective IgA, and lactoferrin, and has been reported to reduce the risk of development of NEC (48–50). HM may also protect against NEC through the presence of epidermal growth factor, which attenuates TLR4 signaling via activation of the phosphoinositide 3-K signaling pathway (51).

## Randomized Controlled Trials and Observational Studies

Due to the role of the intestinal microbiome in the pathogenesis of NEC, dietary supplementation with probiotics to modulate the intestinal microbiome has been proposed as a strategy to reduce the risk of NEC and associated morbidity and mortality. An overview of the characteristics of randomized controlled trials (RCTs) evaluating probiotics, prebiotics, and synbiotics for NEC are shown in Table 1. Of the thirty-four RCTs evaluating probiotics for NEC, seventeen reported significant beneficial effects, eleven reported no health benefit, and six reported a trend to prevent NEC. The Probiotics in Preterm Infants (PiPs) trial, the largest trial to date of a probiotic intervention, assessed the effectiveness of *Bifidobacterium breve* BBG-001 to reduce NEC, late onset sepsis (LOS), and death in 1,315 preterm infants in the UK (60). The trial did not find a significant reduction in NEC and the authors did not recommend the routine use of probiotics in this population. An important limitation to note of this trial was the high rates of cross-colonization in the placebo group which may have confounded the results. The ProPrems RCT compared daily administration of a probiotic mixture containing *Bifidobacterium infantis, Streptococcus thermophilus*, and *Bifidobacterium lactis* with placebo in 1,099 very preterm infants (70). Infants receiving the probiotic mixture had a significantly lower incidence of NEC (stage 2 or greater) compared to control infants. The 2015 ProPre-Save RCT evaluated the efficacy of probiotic alone, prebiotic alone, or combined (synbiotic), on the prevention of NEC in 400 VLBW infants (88). Infants were randomized to either a control group or one of three study groups. The study groups were administered probiotic (*B. lactis*), prebiotic (inulin), or synbiotic (*B. lactis* plus inulin) for up to eight weeks. The probiotic and synbiotic groups had a lower incidence of NEC compared to the prebiotic and control groups. The study groups had reduced mortality, reduced nosocomial sepsis, faster time to reach full enteral feeding, and shorter NICU duration compared to the control group. Another large RCT randomly assigned 750 preterm infants to receive *Lactobacillus reuteri* DSM 17938 or placebo (76). Here, a non-significant 40% decrease in NEC was reported in the probiotic group compared with control group.

**Table 1:** Characteristics of randomized controlled trials evaluating probiotics, prebiotics, and synbiotics for NEC.

**Author**	**Year**	**Country of origin**	**Sample size**	**Participant details**	**Details of intervention, probiotic species, and strains**	**Main finding**
**Probiotic interventions**						
Al-Hosni et al. (52)	2012	USA	101	ELBW	LGG, *B. infantis*	No significant difference in incidence of NEC
Arora et al. (53)	2017	India	150	GA ≤ 34 wk	*S. boulardii, L. rhamnosus, L. acidophilus, B. longum*	Reduced incidence and severity of NEC
Awad et al. (54)	2010	Egypt	150	PT admitted to NICU	*L. acidophilus*, Living and Killed	Reduced incidence of NEC Killed retained similar benefits to live bacteria
Benor et al. (55)	2014	Israel	49	Mothers of VLBW infants	*L. acidophilus* and *B. lactis*	Reduced incidence of NEC and in stage 2 NEC
Bin-Nun et al. (56)	2005	Israel	145	BW ≤ 1,500 g	*B. infantis, S. thermophilus*, and *B. bifidus*	Reduced both incidence and severity of NEC
Braga et al. (57)	2011	Brazil	231	VLBW, BW 750–1,500 g	*L. casei, B. breve*	Reduced incidence of NEC (stage ≥2)
Chandrashekar et al. (58)	2018	India	145	GA <34 wk	*L. acidophilus, L. rhamnosus, B. longum*, and *S. boulardii*	Reduced incidence and severity of NEC
Chowdhury et al. (59)	2016	Bangladesh	119	VLBW, GA 28–33 wk	LGG, *L. paracasei, L. casei, L. acidophilus, L. lactis, B. bifidum, B. longum, B. infantis*	Reduced incidence of NEC
Costeloe et al. (60)	2016	UK	1310	GA 23–30 wk	*B. breve* BBG-001	No evidence of benefit
Dani et al. (61)	2002	Italy	585	GA <33 wk or BW <1,500 g	LGG	Non-significant reduction in incidence of NEC
Dashti et al. (62)	2014	Iran	136	BW 700–1,800 g	*L. acidophilus, L. rhamnosus, L. bulgaricus, L. casei, S. thermophilus, B. longum, B. breve*	No evidence of benefit
Demirel et al. (63)	2013	Turkey	271	BW ≤ 1,500 g GA ≤ 32 wk	*S. boulardii*	No significant difference in incidence of NEC
Dongol-Singh et al. (64)	2017	Nepal	72	Hospitalized PT	*L. rhamnosus* LCR35	Trend toward reduction in incidence of NEC
Fernández-Carrocera et al. (65)	2013	Mexico	150	BW <1,500 g	*L. rhamnosus, L. casei, L. plantarum, L acidophilus, B. infantis, S. thermophilus*	Trend toward reduction in incidence of NEC
Gomez-Rodriguez et al. (66)	2019	USA	90	ELBW and VLBW (700–1,500 g)	*L. acidophilus boucardii* versus mix *of L. acidophilus, L. rhamnosus, L. casei, L. plantarum, B. infantis*, and *S. thermophilus*	No difference between use of single strain or multispecies probiotics on NEC incidence
Hays et al. (67)	2015	France	199	GA 25–31 wk, BW 700–1,600 g	*B. lactis* or *B. longum* or both	Incidence rates of NEC similar in the two groups
Hernández-Enríquez et al. (68)	2016	Mexico	44	GA <34 wk or BW ≤1,550 g	*L. reuteri* DSM *17938*	Reduced incidence of NEC
Hoyos et al. (69)	1999	Colombia	1237	NICU	*L. acidophilus* and *B. infantis*	Reduced incidence of NEC and NEC-associated fatalities
Jacobs et al. (70)	2013	Australia and New Zealand	1099	GA <32 wk, BW <1,500 g	*B. infantis, S. thermophilus, B. lactis*	Reduced incidence of NEC of stage 2 or more
Janvier et al. (71)	2014	USA	611	GA <32 wk, NICU	*B. breve, B. bifidum, B. infantis, B. longum, L. rhamnosus HA-111*	Reduced incidence of NEC
Kaban et al. (72)	2019	Indonesia	94	GA 28–34 wk, BW 1,000–1,800 g	*L. reuteri* DSM 17938	Trend to reduction in incidence of NEC
Lin et al. (73)	2005	Taiwan	367	VLBW	*L. acidophilus, B. infantis* (Infloran®)	Reduced incidence and severity of NEC
Lin et al. (74)	2008	Taiwan	434	VLBW	*B. bifidum, L. acidophilus*	Reduced incidence of NEC
Oncel et al. (75)	2014	Turkey	424	BW ≤ 1,500 g, GA ≤ 32 wk	*L. reuteri* DSM 17938	No significant difference in incidence of NEC
Rojas et al. (76)	2012	Colombia	750	PT, BW ≤ 2,000 g	*L. reuteri* DSM 17938	Non-significant decrease in incidence of NEC
Saengtawesin et al. (77)	2014	Thailand	60	BW ≤ 1,500 g, GA ≤ 34 wk	*L. acidophilus, B. bifidum* (Infloran®)	No difference in incidence of NEC stage ≥2
Samanta et al. (78)	2009	India	186	Very PT or VLBW	*B. infantis, B. bifidum, B. longum, L. acidophilus*	Reduced morbidity due to NEC
Sari et al. (79)	2011	Turkey	221	BW <1,500 g, GA <33 wk	*L. sporogenes*	No significant difference in incidence of death or NEC
Serce et al. (80)	2013	Turkey	208	GA ≤ 32 wk, BW ≤ 1,500 g	*S. boulardii*	Did not decrease the incidence of NEC
Shadkam et al. (81)	2015	Iran	60	GA 28–34 wk, BW 1,000–1,800 g	*L. reuteri* DSM 17938	Reduced incidence of NEC
Shashidhar et al. (82)	2017	India	104	BW ≤ 1,500 g	*L. acidophilus, L. rhamnosus, B. longum, S. boulardii*	Trend toward lower incidence NEC
Van Niekerk et al. (83)	2015	South Africa	184	GA <34 wk and VLBW (<1,250 g)	*Pro-B2: LGG* and *B. infantis*	Reduced incidence of NEC in VLBW but not in HIV-exposed infants
Wang et al. (84)	2014	China	100	FT in NICU	*L. casei, L. acidophilus, B. subtilis* and *E. faecalis*	No significant difference in the incidence of NEC
Zampieri et al. (85)	2013	Italy	32	BW 600–1,500 g with stage 2 NEC	*L. paracasei* subsp. *paracasei* F-19	Reduced clinical progression of NEC
**Prebiotic interventions**						
Armanian et al. (86)	2014	Iran	75	BW ≤ 1,500 g, GA ≤ 34 wk	scGOS/lcFOS	Reduced incidence of NEC
Dasopoulou et al. (87)	2015	Greece	167	PT admitted to NICU	scGOS/lcFOS	No significant difference in incidence of NEC
Dilli et al. (88)	2015	Turkey	400	VLBW	Inulin	No significant difference in incidence of NEC
Manzoni et al. (89)	2009	Italy	743	VLBW	Lactoferrin	Reduced incidence of ≥stage 2 NEC and of death
Riskin et al. (90)	2010	Israel	28	GA 23–34 wk	Lactulose	Fewer episodes of lower stage NEC
**Synbiotic interventions**						
Dilli et al. (88)	2013	Turkey	100	Infants with cyanotic congenital heart disease	*B. lactis* plus inulin	Reduced incidence of NEC
Dilli et al. (91)	2015	Turkey	400	VLBW	*B. lactis* plus inulin	Probiotic alone and synbiotic but not prebiotic alone reduced incidence of NEC
Guney-Varal et al. (92)	2017	Turkey	110	GA ≤ 32 wk and BW ≤ 1,500 g	*L. rhamnosus, L. casei, L. plantarum, B. animalis* plus FOS and GOS	Reduced the incidence of NEC and mortality rate
Manzoni et al. (89)	2009	Italy	743	VLBW	LGG plus lactoferrin	Reduced incidence of ≥stage 2 NEC and of death
Nandhini et al. (93)	2016	India	220	GA 23–34 wk, BW >1,000 g	PREPRO HS®—*L. acidophilus, B. longum, L. rhamnosus, L. plantaris, L. casei, L. bulgaricus, B. infantis*, and *B. breve* plus FOS	Reduced incidence of NEC, did not reduce severity of NEC

*VLBW, very low birth weight; wk, weeks; ELBW, extremely low birth weight; GA, gestational age; BW, birth weight; PT, preterm; FT, full term; GOS, galacto-oligosaccharides; FOS, fructo-oligosaccharides; scGOS, short-chain galacto-oligosaccharides; lcFOS, long-chain fructo-oligosaccharides. Search Strategy: We searched PubMed and CENTRAL in May 2021 with the search terms “premature” or “prematurity” and “probiotic” or “prebiotic” or “synbiotic” and “necrotizing enterocolitis.” Publication type: “clinical trial” and “randomized controlled trial.” The RCT was excluded if only the abstract was available*.

A 2016 retrospective multi-center study examined data from 10,890 preterm infants from 44 NICUs in Germany with routine use of a dual strain probiotic (Infloran™, *Lactobacillus acidophilus* and *B. infantis*) (94). Infloran administration significantly reduced the incidence of NEC, mortality after NEC, overall mortality, and nosocomial bloodstream infection. Subgroup analysis in ELBW infants revealed that these effects were even more pronounced in these infants. Gray et al. performed a multi-center cohort study of 78,076 preterm infants from 289 NICUs in the United States from 1997 to 2016 (95). The most commonly administered probiotic was *Lactobacillus* (71%), followed by Ultimate Flora (*Bifidobacterium* and *Lactobacillus*), ABC Dophilus (*Bifidobacterium, Lactobacillus*, and *Streptococcus*), and Align (*Bifidobacterium*). Probiotic administration increased over time and was associated with a decrease in the incidence of NEC and death. In contrast to other studies reporting that probiotics reduce *Candida* colonization, an increase in *Candida* infection was observed here. The authors state that confirmatory reports are required to determine if the findings are clinically significant. Probiotic use was not associated with an increase in bloodstream infection or meningitis. Concerning the safety of probiotics in preterm infants, their use has very rarely been associated with deleterious side effects such as bacterial sepsis due to probiotic translocation (61, 96, 97). The cost benefits ratio is very much in favor of probiotics considering the data from the numerous preterm infants who have receive such supplementation (98). In Canada, a 2019 retrospective cohort study evaluated the effect of probiotic administration on extremely preterm infants (<29 weeks gestational age) admitted to NICU (99). 3093 infants were included in the analysis with 652 infants receiving probiotic preparations, either Florababy (*B. breve, Bifidobacterium bifidum, B. infantis, Bifidobacterium longum*, and *Lactobacillus rhamnosus* GG) or Biogaia (*L. reuteri*). Probiotic use was associated with a significant reduction in the rate of NEC and mortality but not in the rate of LOS.

## Systematic Reviews and Meta-Analysis

A 2020 Cochrane review of 56 RCTs (*n* = 10,812) compared probiotic supplementation with placebo in very preterm or VLBW infants (100). This review reported that probiotics may reduce the risk of NEC and probably reduces mortality for very preterm or VLBW infants. The evidence for this was assessed as low certainty due to weaknesses in trial design particularly with regards measures used to blind clinicians and caregivers to the intervention. Small-study bias was also a concern with most of the included trials small in size (median *n* = 149). Heterogeneity of the probiotic interventions used in RCTs was reported by the authors as the main challenge in applying the findings of the review. Additionally, the authors noted that few trials provided data for extremely preterm or ELBW infants.

A 2020 systematic review and network meta-analysis (NMA) analyzed data from 63 RCTs (*n* = 15,712) to assess the effectiveness of various single-strain and multi-strain probiotics for the prevention of NEC mortality and morbidity (101). High-certainty evidence indicated that combinations of *Bifidobacterium* and *Lactobacillus* were most effective for the prevention of mortality and stage 2 NEC. Moderate-certainty evidence suggested that *B. lactis, L. rhamnosus*, and *L. reuteri* prevent stage 2 NEC. Moderate-certainty evidence also indicated that *B. lactis* and *L. reuteri* reduced hospital stay. Low-certainty evidence suggested that combinations of *Bacillus* and *Enterococcus*; *Lactobacillus, Bifidobacterium*, and *Enterococcus*; and *Bifidobacterium* and *S. thermophilus* may prevent stage 2 NEC. Important limitations as noted by the authors were the lack of available data comparing the effects of different probiotic strains with each other and the lack of strain level information in many of the trials. Most recently in 2021, a NMA of 51 RCTs (*n* = 10,664) examined the role of probiotics in preventing NEC (102). *L. acidophilus* LB was ranked as the best supplementation option for reducing NEC risk in preterm infants. The NMA also included a subgroup analysis of type of feeding; exclusively HM fed vs. formula fed (alone or combination with HM). The administration of *B. lactis* Bb-12/B94 was associated with a reduced risk of NEC stage ≥2 in exclusively HM-fed infants and non-exclusively HM-fed infants. The relative size effect favored exclusively HM-fed infants.

Chi et al. also employed a NMA approach based on 45 RCTs (*n* = 12,320) to compare probiotic, prebiotic, and synbiotics for premature infants (103). The RCTs included strains of *Bifidobacterium, Lactobacillus, Enterococcus, Streptococcus, Bacillus*, and *Saccharomyces*, alone and in combination. Supplementation with *Bifidobacterium* plus *Lactobacillus* was associated with lower rates of morbidity and mortality in NEC. *Lactobacillus* plus prebiotic was associated with lower rates of NEC morbidity and had the highest probability of having the lowest rate of NEC. *Bifidobacterium* plus prebiotic had the highest probability of having the lowest rate of mortality. The authors found that the efficacy of single strain supplements was limited and recommended the use of synbiotics particularly those including both *Bifidobacterium* and *Lactobacillus*. A limitation of this NMA was the insufficient data available for extremely preterm or ELBW infants. A 2018 meta-analysis used 18 RCTs (*n* = 1,322) to evaluate whether prebiotics alone could reduce the incidence of sepsis, NEC, and mortality in preterm infants (104). Participants who received prebiotics showed significant decreases in the incidence of sepsis and mortality; however, there was no significant differences between intervention and control groups in relation to the morbidity rate of NEC.

## Clinical Practice Guidelines

In 2020, the European Society of Pediatric Gastroenterology Hepatology and Nutrition (ESPGHAN) published a position paper aiming to provide recommendations relating to the use of probiotics in preterm infants (105). A conditional recommendation for the use of *L. rhamnosus* GG ATCC 53103 or the combination of *B. lactis* BB-12, *B. infantis* BB-02, and *S. thermophilus* TH-4 was made. It was advised that strains should be selected based on proven effectiveness and an established safety profile. With regard dosage, similar doses as administered in relevant RCTs were recommended. Due to limitations in currently available data, an optimal start of treatment or total duration was not indicated. The paper highlights that probiotics are typically marketed as nutritional supplements and as a result are loosely regulated. Product safety and quality is therefore of concern especially in a vulnerable population such as preterm infants with immature immune systems. ESPGHAN recommended more stringent controls and that probiotic strains be manufactured according to current Good Manufacturing Practice (cGMP) to ensure strain identity, purity, and viability. The probiotic strains should not include any plasmids containing transferable antibiotic resistance genes and local microbiologists must have the ability to routinely detect probiotic sepsis. The panel also recommended against the use of probiotic strains that produce d-lactate, as their potential risks are uncertain. Also in 2020, the American Gastroenterological Association (AGA) published their clinical practice guidelines on the role of probiotics in the management of gastrointestinal disorders (106). The guidelines conditionally recommended probiotics for the prevention of NEC in preterm infants <37 weeks gestational age and low birth weight. The AGA reported that specific probiotics can prevent mortality and severe NEC (stage 2 or greater), reduce days required to reach full feeds, and decrease the duration of hospitalization. The committee identified significant heterogeneity between studies, variability in the strains studied, and a lack of consistent harms reporting as significant knowledge gaps.

Most recently, a 2021 clinical report by the American Academy of Pediatrics (AAP) recommended against the routine administration of probiotics to preterm infants, particularly those whose birth weight is <1,000 g, for the treatment or prevention of NEC (107). The AAP highlights that probiotic products in the US are classified as dietary supplements and are not subject to approval by the US Food and Drug Administration (FDA). As a result, manufacturers can bypass FDA safety, efficacy, and manufacturing standards. The AAP notes that despite the inconsistent data on their safety and efficacy, probiotics are increasingly given to preterm infants in the US with approximately 10% of extremely low gestational age infants receiving a probiotic preparation while in the NICU. The academy advises that centres using probiotics obtain informed consent from parents after discussing the risks and benefits. They also recommended that centres should conduct surveillance to assess the impact of probiotics on the centres microbiota, which could potentially affect all infants, and should carefully document adverse events, outcomes, and safety.

## Conclusions

There is mounting evidence supporting the use of probiotics to decrease the risk of NEC in preterm infants. Several large RCTs have demonstrated that the relative risk for NEC can be reduced using probiotic formulations. It is important to note that some meta-analyses have reported low to moderate level of certainty about the effects of probiotic supplementation on the risk of NEC and the largest RCT to date found no reduction in NEC incidence following supplementation with a single-strain probiotic. A confounding factor in this RCT was the high rates of cross-colonization found in the placebo group. In addition, not all probiotics used in preventing NEC may be equally effective. Therefore, further carefully designed and conducted large-scale RCTs are necessary to determine optimal strains as well as optimal timing and dosing. Furthermore, detailed information about the study population needs to be included such as type of feeding, antibiotic usage, gender, and ethnicity. Data on the particularly vulnerable extremely preterm infants and ELBW infants is limited and more RCTs focused specifically on these groups are needed. Prebiotic and synbiotic interventions are scarcely investigated in RCTs to date and further trials evaluating their efficacy are required. There are conflicting recommendations from experts as to the administration of probiotics to preterm infants for NEC. Concerns about the safety and purity of commercially available probiotics appears to be the greatest hurdle to overcome in terms of the widespread implementation of probiotics in NICU. Many probiotic products are sold as dietary supplements and are not produced under strict quality control conditions. Probiotics which are licensed as a drug by national regulatory authorities should be recommended.

## Author Contributions

KM: conceptualization, original draft preparation, review, and editing. RPR and CS: conceptualization, supervision, review, and editing. CAR and EMD: review and editing. All authors contributed to the article and approved the submitted version.

## Funding

This work was supported by the Science Foundation Ireland grant number SFI/12/RC/2273-P2.

## Conflict of Interest

The authors declare that the research was conducted in the absence of any commercial or financial relationships that could be construed as a potential conflict of interest.

## Publisher's Note

All claims expressed in this article are solely those of the authors and do not necessarily represent those of their affiliated organizations, or those of the publisher, the editors and the reviewers. Any product that may be evaluated in this article, or claim that may be made by its manufacturer, is not guaranteed or endorsed by the publisher.
